# A case of treatment for pulmonary infection caused by multidrug‐resistant *Acinetobacter baumannii*

**DOI:** 10.1002/rcr2.1420

**Published:** 2024-07-01

**Authors:** Chenxia Guo, Shaohua Xu, Wei Yan

**Affiliations:** ^1^ Respiratory Medicine PeKing University Third Hospital Beijing China

**Keywords:** combination therapy, extensively drug‐resistant *Acinetobacter baumannii*, polymyxin B, pulmonary infection, rifampicin

## Abstract

*Acinetobacter baumannii* is a major pathogen in hospital‐acquired infections notorious for its strong acquired resistance and complex drug resistance mechanisms. Owing to the lack of effective drugs, the mortality rate of extensively drug‐resistant *A. baumannii* pneumonia can reach as high as 65%. This article analyzes a case where a combination of cefoperazone‐sulbactam, polymyxin B, and minocycline with rifampicin successfully treated XDR‐AB pulmonary infection. Combination therapy is effective and has a particular clinical value.

## Graphical abstract

Here, we present a successful case of treating an extensively drug‐resistant *A. baumannii* (XDR‐AB) pulmonary infection through the rotational combination of various antibiotics, ultimately achieving success with the addition of rifampicin. This provides a reference for treating such patients in the real world.

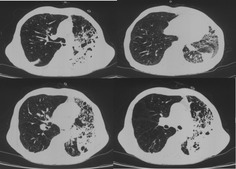

## INTRODUCTION

*Acinetobacter baumannii* (AB) is a gram‐negative, aerobic, rod‐shaped bacterium that has become an important pathogen that causes hospital‐acquired infections worldwide because of its strong acquired resistance, clonal spread, and long‐term survival ability in hospital environments. A large‐scale cross‐sectional survey of hospital‐acquired pneumonia pathogens in China showed that AB was the most common, accounting for 16.2%–35.8% of all pneumonia pathogens.^1^ Extensively drug‐resistant *A. baumannii* (XDR‐AB) and pan‐drug‐resistant *A. baumannii* (PDR‐AB) have high mortality rates due to the scarcity of effective treatments. The mortality rate in patients with XDR‐AB pneumonia can reach up to 65%.^2^ Here, we present a successful case of treating an XDR‐AB pulmonary infection through the rotational combination of various antibiotics, ultimately achieving success with the addition of rifampicin. This provides a reference for treating such patients in the real world.

## CASE REPORT

**FIGURE 1:**
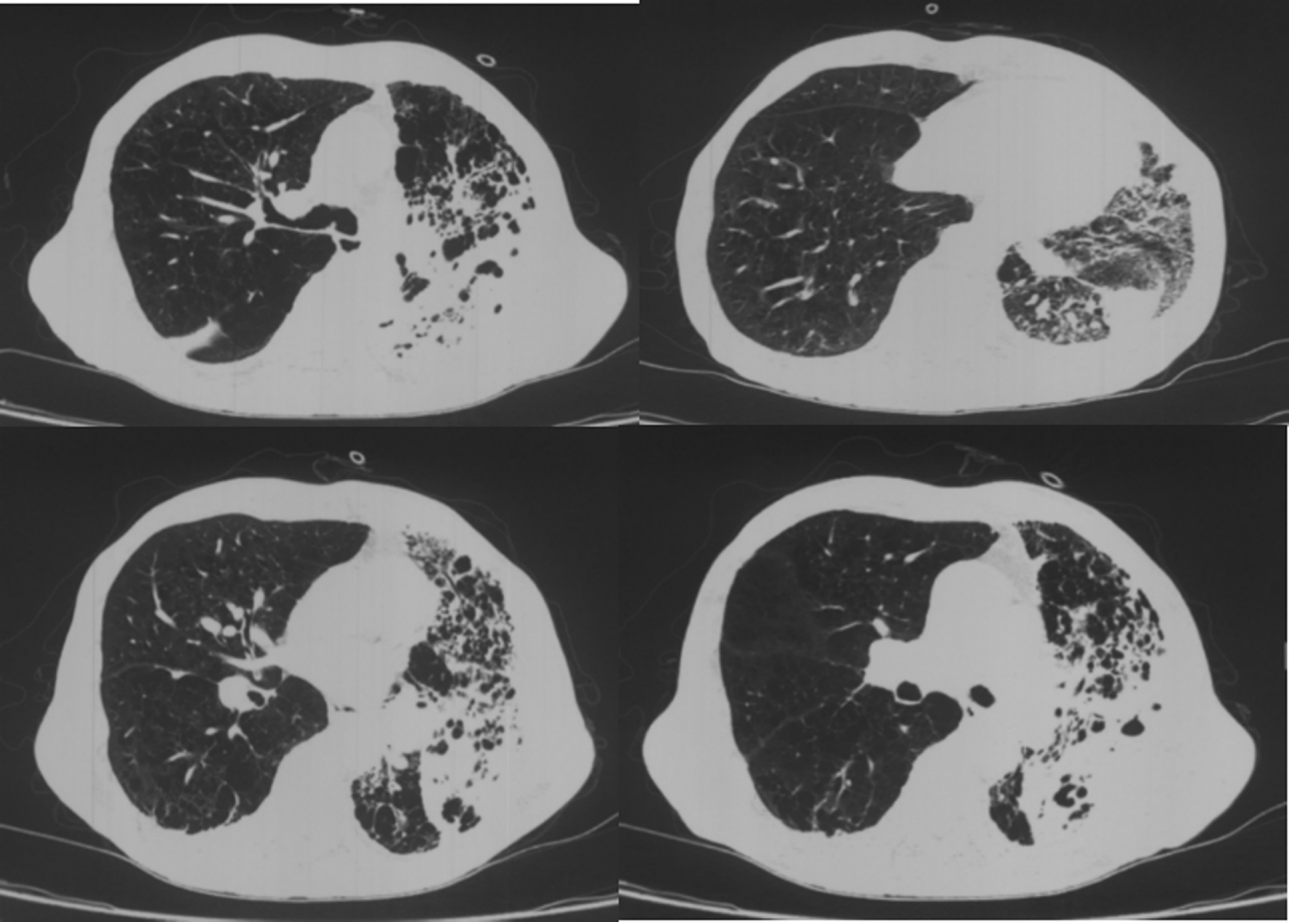
Patchy high‐density shadow in the left lung on chest CT, with an increased extent compared to the previous examination. Bilateral pleural effusion, newly appeared compared to the previous examination. Local narrowing of the left lower lobe bronchus and a suspicious soft tissue mass‐like shadow.

A 68‐year‐old male patient was admitted to the hospital on June 20, 2022, presenting with fever and dyspnea for 10 days. Ten days prior to admission, the patient developed a fever of up to 38°C, without an obvious cause. The fever was accompanied by a cough and yellow or rust‐coloured sputum, and dyspnea worsened with activity. Sputum cultures from a previous hospital revealed methicillin‐resistant *Staphylococcus aureus*. The patient was treated with intravenous cefoperazone‐sulbactam, moxifloxacin, meropenem, and linezolid for anti‐infective therapy, but intermittent fever persisted. He had a previous diagnosis of left lung squamous cell carcinoma, T2aN3M0 stage IIIB with mediastinal and bilateral hilar lymph node metastasis; further, he had received six cycles of chemotherapy and currently receives 200 mg of Toripalimab every 21 days. Additionally, he had a two‐and‐a‐half‐year history of emphysema and bullae and reported experiencing poor lung function (specific details not documented). Upon physical examination, his vital signs were: T 36.1°C, P 102 beats/min, R 34 breaths/min, and BP 90/60 mmHg. The patient exhibited diffuse wheezing sounds in the left lung and moist rales in the lower left lung. Auxiliary examination: Routine blood (June 10, 2022): white blood cell count (WBC) 7.5 × 10^9^/L, neutrophil ratio (NE%) 89.9%, and procalcitonin (PCT) 50.53 ng/mL. Chest CT taken on June 16, 2022 revealed patchy high‐density shadows in the left lung with an enlarged range, as well as new appearances of bilateral pleural effusion. Local narrowing of the left lower lobe bronchus and a suspected soft tissue mass shadow were observed (Figure 1).

Upon admission, based on the previous sputum culture results, the patient was treated with intravenous imipenem/cilastatin (0.5 g q8h) in combination with linezolid (0.6 g q12h) for anti‐infective therapy, and sputum and blood cultures were promptly sent. Bronchoscopy was performed, and a large amount of yellow purulent secretion was suctioned out and sent for culture and next‐generation sequencing (NGS) of sputum DNA. The sputum culture and drug sensitivity report showed *A. baumannii*, which was resistant to meropenem (MIC ≥16 ug/mL), imipenem (MIC ≥16 ug/mL), cefoperazone/sulbactam (MIC ≥64 ug/mL), piperacillin/tazobactam (MIC ≥128 ug/mL), and levofloxacin (MIC ≥8 ug/mL), but sensitive to minocycline (MIC 4 ug/mL), tigecycline (MIC 2 ug/mL), and polymyxin B (MIC 2 ug/mL). NGS revealed 967,771 sequences of *A. baumannii*, 1,544,400 sequences of *S. aureus*, and 50,799 sequences of *Pseudomonas aeruginosa*. In addition, the resistance genes OXA‐23 (453 sequences), mecA (1463 sequences), and OXA‐66 (418 sequences) were detected, suggesting dual infection with XDR‐AB and methicillin‐resistant *S. aureus* (MRSA).

After admission, the patient had a persistent fever, with his temperature fluctuating between 37.8 and 38.5°C. According to the antibiotic sensitivity test, the four antibiotics to which *A. baumannii* is sensitive are minocycline (MIC = 4), sulfamethoxazole/trimethoprim (MIC<20), tigecycline (MIC = 2), and colistin (MIC<0.5).The antibiotic regimen was adjusted to imipenem/cilastatin (0.5 g q8h, intravenous infusion) combined with minocycline (100 mg q12h, oral) for XDR‐AB coverage and linezolid (0.6 g q12h, intravenous infusion) for MRSA coverage. However, the patient developed an urticarial rash on both upper limbs and dyspnea after treatment, suggesting a possible drug allergy. Therefore, minocycline was discontinued, and symptomatic anti‐allergic treatment was administered. The regimen was changed to imipenem/cilastatin (0.5 g q8h, intravenous infusion) combined with tigecycline (50 mg q12h, intravenous infusion) for XDR‐AB coverage. During infusion of imipenem/cilastatin, the patient developed a rash again, suggesting a possible allergy to this combination of drugs. Therefore, the treatment regimen was switched to meropenem (1 g q8h, intravenous infusion).

**FIGURE 2:**
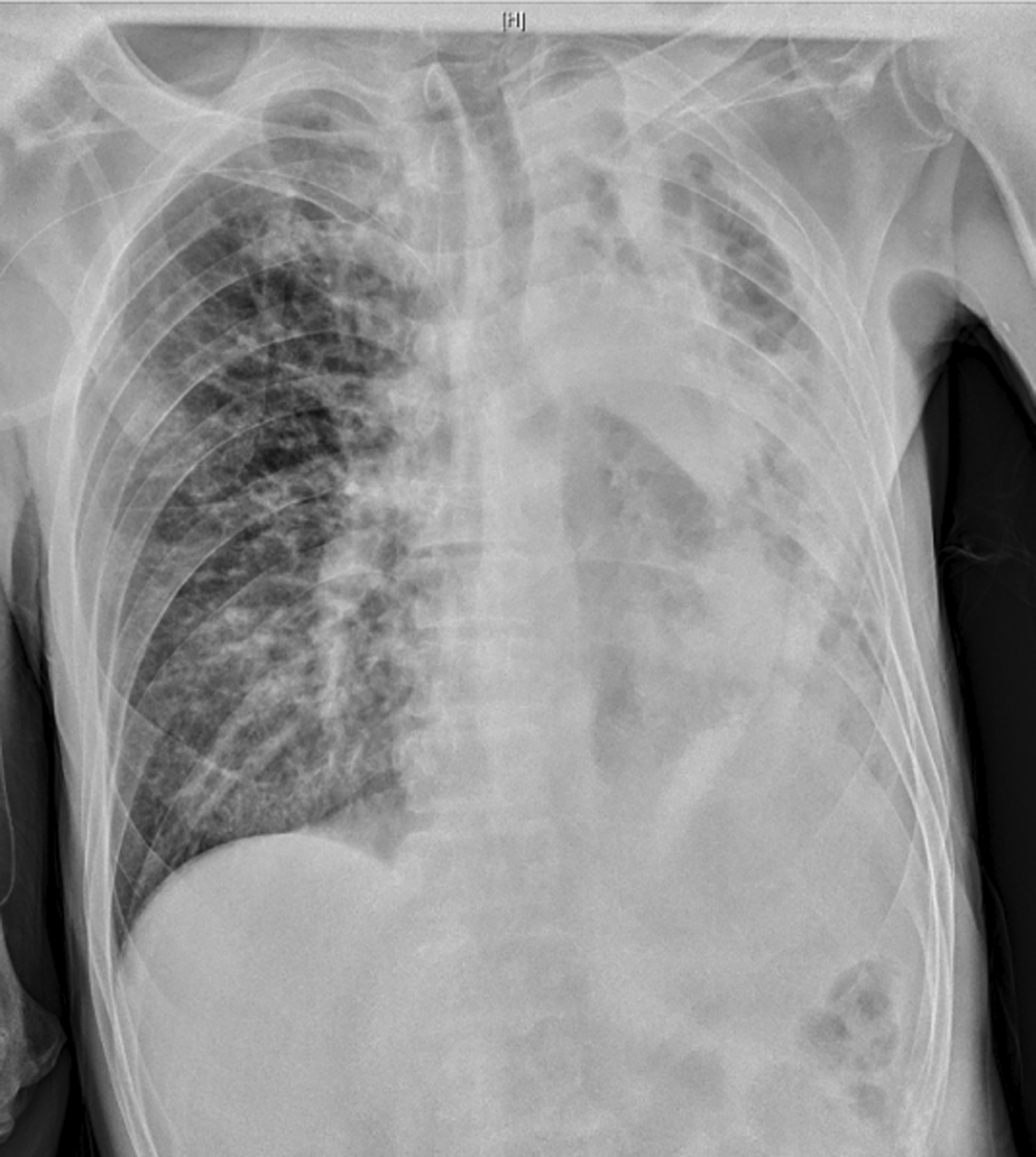
Chest x‐ray follow‐up on July 3rd showing an increased infiltrative lesion in the left lung compared to the previous examination.

On the ninth day after admission, the patient's temperature began to decrease, with a reduction in cough frequency, sputum volume, and dyspnea. However, the patient's temperature remained elevated, fluctuating between 37.0 and 37.8°C. Therefore, the treatment regimen was changed to polymyxin B (50 WU q12h, intravenous infusion) and meropenem (1 g q8h, intravenous infusion). On the fourteenth day after admission, the patient's temperature increased to 38.4°C, and the chest X‐ray showed an increased left lung infiltrate compared to before (Figure 2). The sputum culture still showed XDR‐AB, and the regimen was changed to piperacillin/tazobactam (5 g q8h, intravenous infusion) and polymyxin B (50 WU q12h, intravenous infusion) as anti‐infective therapy. The patient's temperature continued to rise, up to a maximum of 38°C, and dyspnea worsened.

**FIGURE 3:**
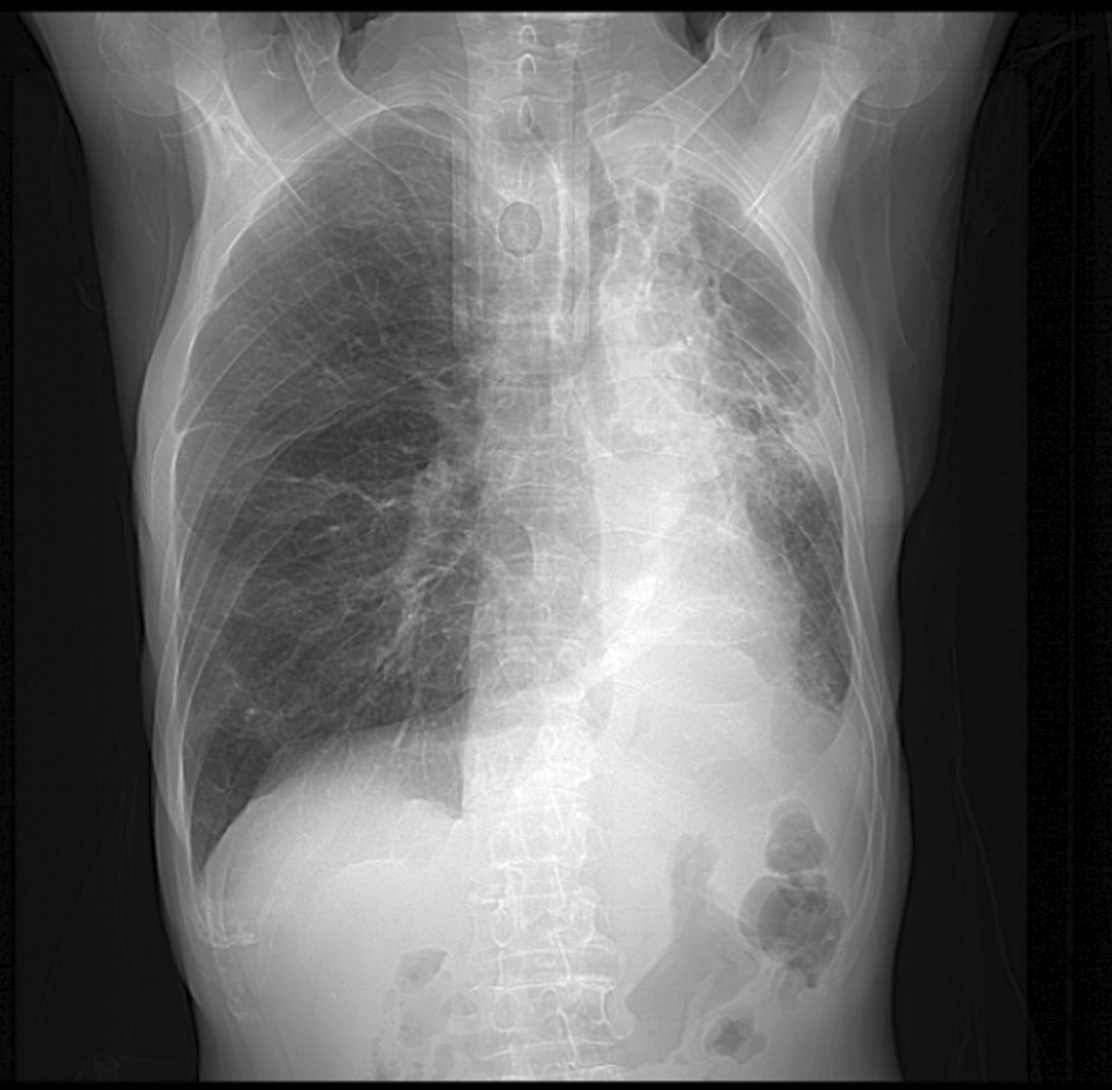
Chest x‐ray follow‐up on August 19th showing significant absorption of the infiltrative shadow in the left lung compared to the previous examination.

On the nineteenth day after admission, piperacillin/tazobactam was discontinued and replaced with cefoperazone/sulbactam (3 g q6h, intravenous infusion), and rifampicin (0.6 g q24h, oral) was added, in combination with polymyxin B (50 WU q12h, intravenous infusion) and oral minocycline (100 mg q12h) for anti‐infective therapy. Three days later, the patient's temperature returned to normal, and his coughing and sputum production significantly reduced. A follow‐up chest radiography showed significant improvement in the left lung infiltrates (Figure 3). Follow‐up chest radiography revealed improved pneumonia in both lungs, and the patient was eventually discharged. After discharge, the patient did not experience fever.

## DISCUSSION

*A. baumannii* (AB) is a frequent cause of hospital‐acquired pneumonia, primarily targeting the lungs. Resistance rates of this pathogen to cefoperazone/sulbactam and minocycline were 49.7% and 38.8%, respectively. However, its resistance rates to polymyxin B and tigecycline were relatively low (0.7% and 5.0%, respectively), whereas the resistance rates to other tested drugs were mainly above 40%.^3^

Initially, we chose tigecycline in combination with carbapenems for treatment; however, the effect was unsatisfactory. NGS was performed on the patient's sputum, and the results showed the presence of XDR‐AB along with the resistance gene OXA‐23. The mechanisms of AB resistance are diverse, and OXA‐23 can produce carbapenemases that exhibit strong hydrolytic activity against imipenem and meropenem in vitro. The resistance mechanism may therefore be the main underlying cause for the patient's poor initial treatment response to a regimen containing carbapenems.

The number of antibiotics effective against MDRAB (Multidrug‐resistant *A. baumannii*) is limited, and polymyxins serve as the cornerstone of treatment. However, the use of polymyxins alone often fails to achieve satisfactory therapeutic effects. Current in vitro experimental models have confirmed that combinations with other antibiotics, such as rifampicin, imipenem, sulbactam, and tetracycline, can achieve synergistic therapeutic effects,^4^, ^5^, ^6^ a result that was confirmed by the treatment of our patient. Relying solely on antibiotic susceptibility results may not always yield good outcomes in real cases; factors such as the patient's immune status, drug side effects, and bacterial resistance mechanisms can all influence treatment outcomes. The successful treatment of our patient with the combination of rifampicin and polymyxin B may be attributed to the synergistic effects of rifampicin. Apart from interfering with RNA synthesis based on the effect of polymyxin B on the bacterial outer membrane, rifampicin has synergistic effects with polymyxin B, shortening the microbial clearance time and increasing the clearance rate. Further, it can synergistically disrupt biofilms formed by AB with tetracycline antibiotics, thus counteracting AB's high resistance.^7^, ^8^, ^9^ Finally, the treatment regimen was adjusted to polymyxin B in combination with cefoperazone/sulbactam, minocycline, and rifampicin, which proved effective.

Currently, there is a lack of large‐sample clinical data for selecting the optimal treatment regimen for MDR A. baumannii pulmonary infections. Combination therapy using sulbactam, polymyxin B, minocycline, or rifampicin is an effective treatment option. However, antibiotics should be selected reasonably based on the patient's condition and laboratory test results to avoid the spread of highly resistant or multidrug‐resistant strains. The advantage of this antibiotic combination is that the combined use of multiple antibiotics can exert the synergistic effect of multiple antibacterial drugs and improve the clearance rate of microorganisms, which has clinical advantages over the single drug treatment of AB infection. The disadvantage is that the adverse reactions to drugs, including their impact on liver and kidney function, need to be closely monitored.

## AUTHOR CONTRIBUTIONS

ChenXia Guo was the treating physician and first author of this article. ShaoHua Xu discussed diagnostic methods and treatment strategies. Wei Yan supervised the clinical practice and revised the manuscript.

## FUNDING INFORMATION

Supported by Beijing Municipal Natural Science Foundation, 7212128.

## CONFLICT OF INTEREST STATEMENT

None declared.

## ETHICS STATEMENT

The authors declare that a written informed consent was obtained for the publication of this manuscript and the accompanying images. This study was approved by the Ethics Committee of Peking University Third Hospital.

## Data Availability

Data sharing not applicable—no new data generated.
